# Aggressive skeletal muscle metastasis from cervical cancer invading into the spinal canal: A case report

**DOI:** 10.1002/ccr3.1952

**Published:** 2019-01-15

**Authors:** Ai Miyoshi, Yoshihiro Kuritani, Serika Kanao, Hirokazu Naoi, Hirofumi Otsuka, Takeshi Yokoi

**Affiliations:** ^1^ Department of Obstetrics and Gynecology Kaizuka City Hospital Kaizuka Japan

**Keywords:** skeletal muscle metastasis, spinal canal, spinal invasion, uterine cervical cancer

## Abstract

We present the first case of the patient with skeletal metastasis of uterine cervical cancer which invaded the vertebral body and spinal canal, with consequent paralysis of the lower extremities.

## 1 INTRODUCTION

We report on a rare case of aggressive skeletal metastasis of a uterine cervical cancer occurring 25 months after initial treatment. The metastatic tumor, detected in the latissimus dorsi, was resected once. However, a tumor recurrence invaded the vertebral body and spinal canal, with consequent paralysis of the lower extremities.

Paradoxically, the incidence of cancer metastasis to skeletal muscle is reported to be less than 2% of all metastases, despite that skeletal muscle accounts for nearly 50% of total body mass and is characterized by a rich blood supply.^1^ The clinical outcome of patients with muscle metastasis has been reported to be poor .^1^, ^2^ To our knowledge, a metastasis from uterine cervical cancer to a skeletal muscle, which subsequently invaded into the spinal bone and canal, has not been previously reported. We now report on such a case, an aggressive cervical to skeletal metastasis that led to spinal body and spinal canal invasion, with subsequent paralysis of both legs.

## 2 CASE PRESENTATION

A 57‐year‐old woman, suffering from abnormal genital bleeding, consulted her gynecologist. At her first consultation, a cervical tumor, suspected of being a cervical cancer, was detected. She was referred to our hospital for medical treatment of the tumor.

We recognized the easily bleeding tumor in her uterine cervix. Transvaginal ultrasonography showed a tumor, 3 cm in diameter. The uterine corpus and both ovaries were normal in appearance. Pelvic magnetic resonance imaging (MRI) showed an enhanced cervical tumor and a swollen lymph node in the right obturator space. A cervical biopsy revealed a squamous cell carcinoma. The patient was diagnosed as having early‐stage cervical cancer.

We undertook a radical hysterectomy with bilateral salpingo‐oophorectomy and removal of the pelvic lymph nodes. The pathologic diagnosis was of a squamous cell carcinoma of the uterine cervix (non‐keratinizing type), with parametrial invasion and with right obturator lymph node metastasis (pT2bN1M0). We administered concurrent adjuvant chemoradiation (whole pelvic 50.4 Gy/28fr + weekly CDDP, 40 mg/m^2^).

**Figure 1:**
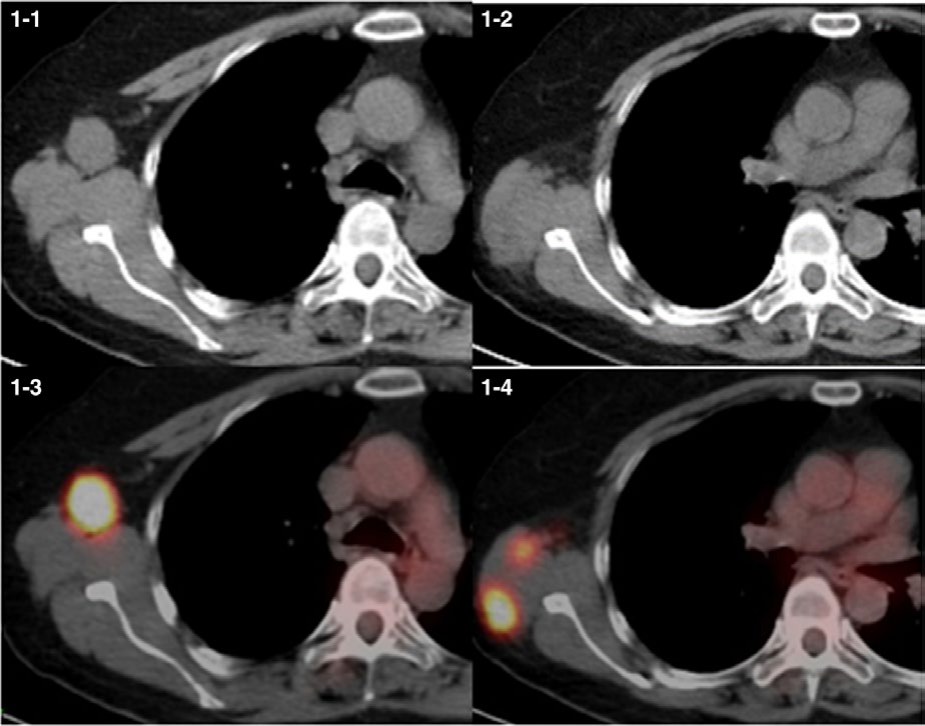
Thoracic CT and FDG‐PET images 26 months after surgery There are metastatic tumors (2.5 cm in diameter) in the right latissimus dorsi muscle (1‐1) and (2 cm in diameter) in the armpit (1‐2). FDG‐PET scan shows uptake in the same areas (1‐3, 1‐4)

**Figure 2:**
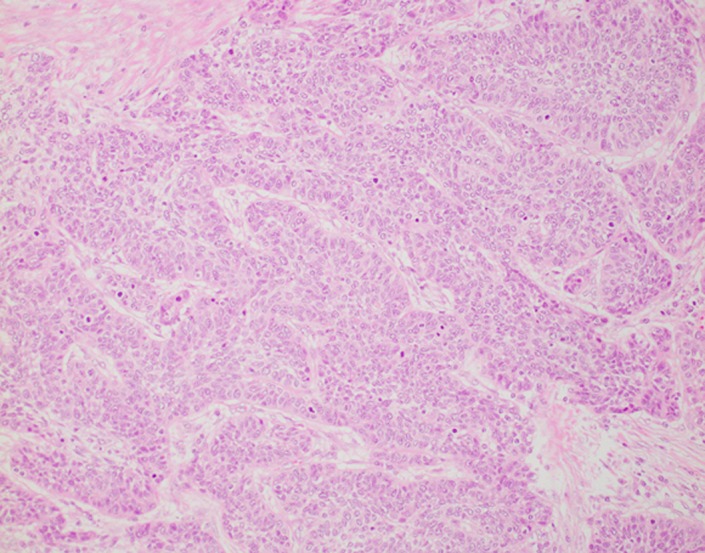
Histological findings of surgical specimen of primary cervical cancer (HE stain; the original magnification is ×200). The tumor is invading to cervical stroma, presenting desmoplastic reaction. The tumor cell shows pleomorphism and the nuclei are hyperchromatic with coarse, granular chromatin. There is no prominent keratinization and no cancer pearls. The histological diagnosis of the primary cervical cancer was a squamous cell carcinoma of non‐keratinizing type

**Figure 3:**
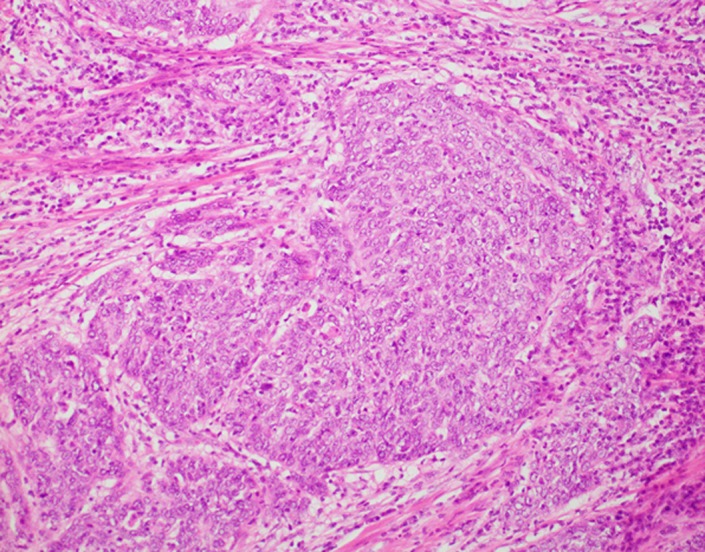
Histological findings of biopsy specimen of the right armpit (HE stain; the original magnification is ×200). The tumor cells show size variation and pleomorphic shapes. The N/C ratio is variable from cell to cell. Chromatin is coarse, granular, and distributed throughout the nuclei with hyperchromatic appearance. The histology of the biopsy specimen is similar to primary cervical cancer. We finally diagnosed the tumor as the metastasis from cervical cancer

At 26 months after the surgery, a follow‐up computed tomography (CT) scan revealed a tumor, 2.5 cm in diameter, in her right latissimus dorsi muscle, and another mass, 2 cm in diameter, in the armpit. Fluorodeoxyglucose‐positron emission tomography (FDG‐PET) imaging showed increased uptake values only in these two tumors and excluded other detectable sites of metastasis (Figure 1). The patient was asymptomatic. However, referring the CT images, we examined her and a tumor at the right armpit was palpated. A needle biopsy of the armpit tumor proved it to be a squamous cell carcinoma (non‐keratinizing type). Histologically, resembling the primary squamous cell carcinoma (Figure 2), the biopsy specimen shows pleomorphic tumor cells with hyperchromatic nuclei (Figure 3). We diagnosed a recurrence of the cervical cancer and, after chemotherapy reduce the size of tumors, planned to perform surgical resection.

Two cycles of combination chemotherapy with Paclitaxel and Carboplatin failed to reduce the size of tumors; however, we still performed the surgical resection. Final pathology of the resected tumors concluded that they were metastases of a squamous cell carcinoma, with similar morphology to the primary disease.

The surgical margins were free of disease. We administered adjuvant radiotherapy to her right latissimus dorsi muscle (50 Gy/25Fr). In a follow‐up CT scan, four months after completion of radiotherapy, we found two recurrent tumors, one (2 cm in diameter) in her right biceps muscle and the other (1 cm in diameter) in the right latissimus dorsi muscle. FDG‐PET showed increased uptake values in these two tumors and excluded other sites of metastasis. We again performed surgical resection. Metastatic squamous cell carcinoma was again diagnosed. It was pathologically unclear as to whether malignant tissues remained at the resection margins.

Two months later, the patient was admitted suffering a pain in her right biceps muscle, which was accompanied with red swelling and tenderness. CT scan revealed the recurrence of disease in the right latissimus dorsi, thoracic wall, pleura, mediastinal lymph nodes, and in the mediastinum and costal bones. Two regimens of chemotherapy (intravenous nedaplatin and oral etoposide) were administered; neither was effective.

**Figure 4:**
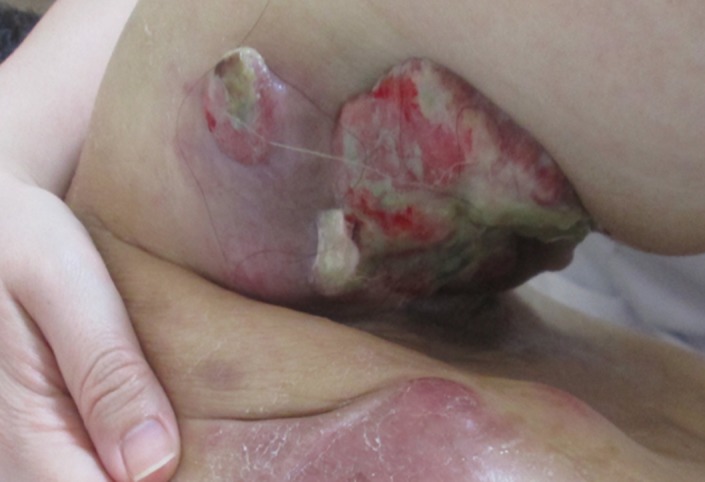
Gross image of the recurrent tumor on right upper arm. The metastatic tumor in the right biceps muscle grew and was exposed on the surface of the skin

**Figure 5:**
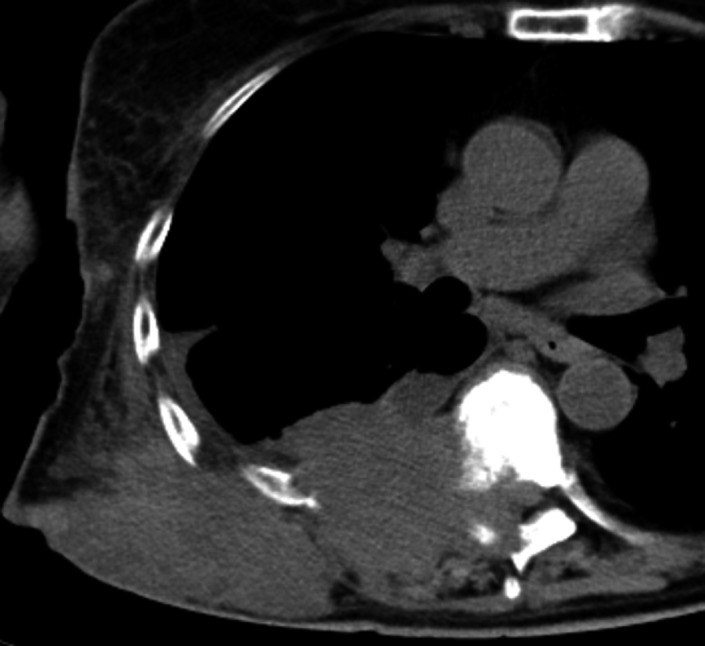
Thoracic CT image of patient with lower extremities paralysis. The tumor in the right latissimus dorsi invaded into the T7 vertebral body and spinal canal and has caused paralysis of lower extremities

The metastatic biceps muscle tumor grew and became exposed on the surface of the skin (Figure 4). She received best supportive care (BSC). Four months after ceasing chemotherapy, she came to our hospital suffering from paralyzed legs. CT revealed that the tumor in the latissimus dorsi invaded into the T7 vertebral body and spinal canal (Figure 5), causing the consequent paralysis of her lower extremities. The patient continued to receive BSC at our hospital; she died of disease two months later.

## 3 DISCUSSION

The metastasis of the cancer to skeletal muscle is exceptionally rare, despite that skeletal muscle accounts for nearly 50% of total body mass and is characterized by a rich blood supply; its incidence has been reported to be less than 1%‐2% of all metastasis .^1^, ^3^ The reasons for its rarity are unclear, although several hypotheses regarding the unique microenvironment of muscle tissue have been proposed. Contractile movement of the skeletal muscle and constant fluctuations in intramuscular blood pressure may act as mechanical barriers for tumor implantation. Obviously, the paracrine microenvironment of muscle tissue is different from more frequent metastases targets. Biochemically, the local production of lactic acid, with a relative lack of oxygen on occasion, could prevent tumor cell survival and proliferation. On the other hand, we can postulate that the abundance of vascularization and rich oxygen levels (when at rest), and rapid fluctuations during exercise, for which the tumor cell is unable to adapt to, could also be anti‐tumorigenic, as shown in hyperbaric oxygen experiments.^4^, ^5^

Protease inhibitors located in the skeletal muscle cell basement membrane may play a vital role in preventing invasion of tumor cells as well. Immune‐mediated antitumor activity by lymphocytes and natural killer cells within the skeletal muscle are other postulated theories that may help reduce the incidence of metastasis.^1^

Surov*et al. *reported that, out of 5170 patients with metastasized cancers identified by CT scan, examined and treated at their institution, only 61 (1.2%) had muscle metastases. The most frequent primary malignancies with metastases into skeletal musculature were genital tumors (24.6% of all muscle metastases), especially cervical cancers (4.9% of 80 lesions). They also reported that the most common skeletal muscle targets for metastases were the iliopsoas (25%), paravertebral (25%), gluteal (16.3%), lower extremity (12.5%), and abdominal wall muscles (10%). Most skeletal muscle metastases were located in the proximal muscle.^3^ As Surov *et al *described it, because of the rarity of skeletal muscle metastasis, most (76%) metastases to muscle were asymptomatic at diagnosis and seem to have been only coincidentally found by CT scans; only 24% were symptomatic.^3^

For the detection of skeletal muscle metastasis, there currently seems to be no other appropriate routine examination than follow‐up routine CT scan. The present case might have been found by a routine CT scan after the primary treatment. During follow‐ups for cervical cancer, we encourage that routine CT scans should be done, covering at least the proximal muscles. A confirmation of cancer metastasis to skeletal muscle, for distinguishing it from a soft tissue sarcoma, needs to have a direct biopsy of the mass.

Skenderi et al also reported that the actual frequency of the skeletal muscle metastases of cervical carcinoma may be underreported for several reasons including their presentation, the lack of clinical symptoms (eg, pain or mass), and clinically undetectable metastatic deposits. Hence, biopsy with morphologic and immunohistochemical assessment should be performed in patients with a previous history of malignancy when these present as a symptomatic, painful mass at unusual locations.^6^

The clinical outcome of patients with rare skeletal muscle metastasis has been reported to be very poor. This is likely due to the presence elsewhere of more diffuse, occult metastases.^1^, ^2^ Reports on treatments for the skeletal muscle recurrence are equally limited. There is as yet no established treatment procedure, and palliative care is often all that is given to these patients. However, Varadarajan *et al *reported that one of their patients, found with an isolated skeletal muscle recurrence as a palpable mass in the left forearm, was put into remission with concurrent chemoradiotherapy; the patient has subsequently obtained 24 months of disease‐free survival. They maintain that, for a solitary distant skeletal muscle recurrence, aggressive therapies, which include surgical resection, with/without concurrent radiation, and with/without concurrent chemotherapy, can be effectively applied.^7^ Saadi *et al *and Ferrandina *et al *also reported on patients with isolated skeletal muscle recurrences treated with surgical resection with radiotherapy, with/without concurrent chemotherapy.^8^, ^9^ These patients were reported to have reached disease‐free status, which has been maintained for 30 months and two months, respectively.

In the present case, we performed surgical resections of the first recurrent lesions because the recurrent site was regional, and because the recurrent tumors were refractory to our cisplatin‐based chemotherapy. Because of the chemoresistance of the recurrent tumor, after the surgical resection we administered adjuvant radiotherapy. Unfortunately, even though the recurrent lesions were pathologically completely resected, the cancer was so aggressive that the next recurrences occurred only four months after the adjuvant therapy. New lesions and symptoms followed, one after another, within several months. Finally, the tumor invaded into the vertebral body and the spinal canal, resulting eventually in lower paralysis and mortality.

Metastasis to skeletal muscle is very rare, and it is generally accompanied by a poor prognosis. The selection of treatment for skeletal muscle metastases is still controversial; however, rather than a palliative approach, multimodal treatments for regional lesions should be considered.

## CONFLICT OF INTEREST

None declared.

## AUTHOR CONTRIBUTION

AM: concepted, designed, and drafted the manuscript. YK, EY, SK, KM, HN, and HO: involved in acquisition of data. TY: supervised the manuscript.
